# Protective Features of Autophagy in Pulmonary Infection and Inflammatory Diseases

**DOI:** 10.3390/cells8020123

**Published:** 2019-02-03

**Authors:** Kui Wang, Yi Chen, Pengju Zhang, Ping Lin, Na Xie, Min Wu

**Affiliations:** 1 West China School of Basic Medical Sciences & Forensic Medicine, and State Key Laboratory of Biotherapy, Sichuan University, and Collaborative Innovation Center for Biotherapy, Chengdu 610041, China; wangkui416@163.com (K.W.); zhangpengju526@163.com (P.Z.); 2 Department of Biomedical Sciences, University of North Dakota, Grand Forks, ND 58203, USA; bio_lp@126.com; 3 Department of Gastrointestinal Surgery, State Key Laboratory of Biotherapy, Sichuan University, Chengdu 610041, China; toddychan@163.com; 4 Section of Infection and Immunity, Herman Ostrow School of Dentistry, Norris Comprehensive Cancer Center, University of Southern California, Los Angeles, CA 90089-0641, USA

**Keywords:** Autophagy, inflammation, acute lung injury, idiopathic pulmonary fibrosis, COPD, tuberculosis, PAH, cystic fibrosis

## Abstract

Autophagy is a highly conserved catabolic process involving autolysosomal degradation of cellular components, including protein aggregates, damaged organelles (such as mitochondria, endoplasmic reticulum, and others), as well as various pathogens. Thus, the autophagy pathway represents a major adaptive response for the maintenance of cellular and tissue homeostasis in response to numerous cellular stressors. A growing body of evidence suggests that autophagy is closely associated with diverse human diseases. Specifically, acute lung injury (ALI) and inflammatory responses caused by bacterial infection or xenobiotic inhalation (e.g., chlorine and cigarette smoke) have been reported to involve a spectrum of alterations in autophagy phenotypes. The role of autophagy in pulmonary infection and inflammatory diseases could be protective or harmful dependent on the conditions. In this review, we describe recent advances regarding the protective features of autophagy in pulmonary diseases, with a focus on ALI, idiopathic pulmonary fibrosis (IPF), chronic obstructive pulmonary disease (COPD), tuberculosis, pulmonary arterial hypertension (PAH) and cystic fibrosis.

## 1. Introduction

Macroautophagy (henceforth referred to as autophagy) is an evolutionarily conserved process by which intracellular materials are sequestered by double-membrane autophagosomes and then delivered to lysosomes for degradation and recycling in various physiological and pathological conditions [1]. The degradation substrates include aggregate–prone proteins, lipids, organelles (including mitochondria, endoplasmic reticulum, peroxisomes, etc.), and intracellular pathogens (bacteria and viruses) [2,3]. The digestion of these autophagy cargoes can maintain cellular homeostasis by facilitating the quality control of the cytoplasm, recycling intracellular components (amino acids, fatty acids, and carbohydrates) to fuel anabolic pathways and energy generation, or by enabling pathogen clearance [2,4]. Therefore, the autophagy process appears to serve as a cellular protective mechanism to counter diverse diseases, including cancer, neurodegenerative diseases, and infectious diseases [3,5]. Conversely, dysregulation of autophagy is also known to exacerbate the disease progression under certain conditions, suggesting that the role of autophagy in human diseases is complex [3,5].

The lung is the primary organ for gas exchange, delivering O_2_ from the atmosphere to the bloodstream and releasing CO_2_ into the atmosphere. During respiration, the lung is continuously exposed to various harmful environmental stimuli, including pathogens (such as viruses and bacteria) and xenobiotics (such as cigarette smoke and particles) [6]. Acute or chronic exposure to these harmful agents can cause damage to the lung, resulting in respiratory dysfunction and pulmonary diseases [6,7]. Both acute lung injury (ALI) and chronic pulmonary diseases are associated with high morbidity and mortality with limited effective therapeutics, thus representing major public health problems worldwide [7,8]. In coping with these outside threats, the lung has evolved various defense mechanisms (such as innate and adaptive immune responses) to maintain its normal function. During the past decade, altered autophagy phenotypes in lung cells have been observed in response to these harmful stimuli [9]. Autophagy is capable of eliminating pathogens, degrading damaged organelles, and regulating inflammatory responses or apoptosis. Thus, autophagy is primarily characterized as a novel defense mechanism for lung injury [10,11,12]. However, autophagy dysfunction has also been reported to represent a harmful event that promotes the progression of pulmonary diseases [11,12]. This seemingly contradictory role of autophagy in pulmonary diseases underlies the lack of an in-depth understanding of the complex autophagy mechanisms in lung injury and pulmonary diseases. In this review, we will summarize the current knowledge of the protective features of autophagy in pulmonary infection and inflammatory diseases, and discuss the perspective of targeting autophagy for the clinical intervention for lung diseases.

## 2. Molecular Regulation of the Autophagy Process

**Figure 1:**
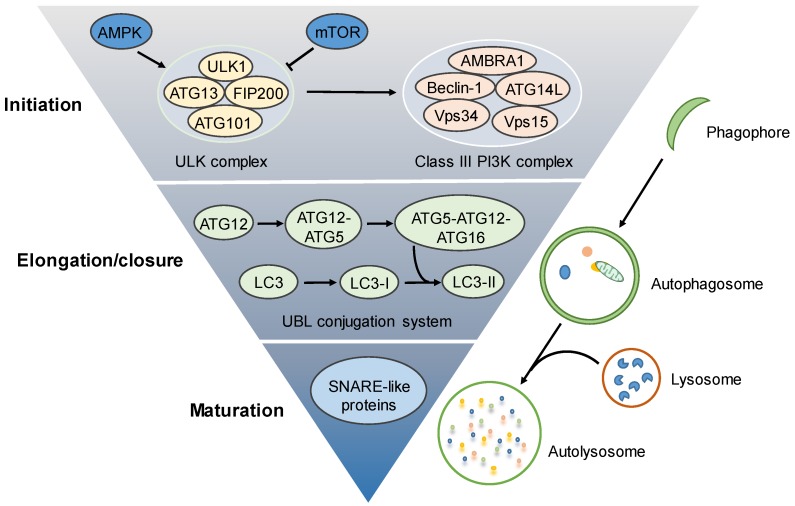
Autophagy machinery. The autophagy process involves initiation, elongation/closure and maturation. The autophagy process is initiated by autophagosome biogenesis to form phagophore, which is regulated by the activation of the preinitiation complex (also known as the ULK complex, containing ULK1/2, ATG13, FIP200, and ATG101) and subsequent activation of the initiation complex (also called the class III PI3K complex, consisting of VPS34, VPS15, Beclin 1, ATG14L, and AMBRA1). The phagophore is then elongated and closed to form a double-membrane autophagosome, which is tightly regulated by the ubiquitin–like (UBL) conjugation systems. The autophagosome will fuse with a lysosome to form an autolysosome for degradation. The SNARE-like proteins may play important roles in autophagosome–lysosome degradation. AMPK—5′-AMP-activated protein kinase; mTOR—the mammalian target of rapamycin; ULK1—UNC51-like kinase 1; class III PI3K—the class III phosphatidylinositol-3-kinase.

The autophagy process involves a sequence of molecular events, including initiation (the formation of phagophore), elongation/closure (the formation of autophagosome), and maturation (the fusion of autophagosome with lysosome) [5] (Figure 1). The initiation of autophagosome biogenesis is triggered by the activation of the UNC51-like kinase (ULK) complex (also called the preinitiation complex), which is composed of ULK1/2, ATG13, FIP200, and ATG101 [4]. The ULK complex can be activated by inactivation of the mammalian target of rapamycin complex 1 (mTORC1), in response to nutrient starvation or the activation of 5′-AMP-activated protein kinase (AMPK) under energy-deprived conditions, to transmit stress signals for autophagosome formation [13]. Meanwhile, in addition to AMPK and mTORC1, the activity of the ULK complex can be regulated by other signals [4]. In turn, the activation of the ULK complex stimulates the class III phosphatidylinositol-3-kinase (class III PI3K) complex (also termed the VPS34 complex or initiation complex), which consists of VPS34, VPS15, Beclin 1, ATG14L, and AMBRA1 [14]. The dissociation of Beclin 1 from Bcl-2/xL anti-apoptotic proteins is a prerequisite for the formation and activation of the class III PI3K complex [14]. This class III PI3K complex enables the conversion of phosphatidylinositol to generate phosphatidylinositol-3-phosphate (PI3P) required for the nucleation of phagophore [15].

The phagophore then elongates and closes up to form the double-membrane autophagosome, which is tightly regulated by PI3P-binding proteins, such as the WD-repeat protein interacting with phosphoinosides (WIPI) family of proteins and two ubiquitin-like (UBL) protein conjugation systems. The completion of the first ubiquitin–like conjugation system leads to the formation of the ATG12/ATG5/ATG16L1 complex, which marks the sites of autophagosome formation and acts as a E3-like ligase for the second conjugation system to generate LC3-II (LC3-PE, the phosphatidylethanolamine-conjugated LC3) from the ATG4–mediated proteolytic cleavage of LC3 [4,16]. The lipidated LC3-II is closely associated with the elongation of the phagophore for autophagosome formation [17]. Following the completion of autophagosome formation, the autophagosome will fuse with a lysosome to form an autolysosome, in which the sequestered contents are degraded by a variety of lysosomal acid hydrolases and released into the cytosol for recycling [18,19].

Autophagy-mediated degradation was previously recognized as a nonspecific process to remove cellular debris. Recently, increasing evidence suggests that autophagic encapsulation and degradation in some cases could be highly selective for specific substrates (termed ‘selective autophagy’) [2,3]. For example, autophagy can selectively digest damaged or depolarized mitochondria for the maintenance of mitochondrial homeostasis (mitophagy), remove invading pathogens (i.e., bacteria) to enhance host defense (xenophagy), clear polyubiquitinated protein for protein turnover (aggrephagy), and so on [12,20,21]. These types of selective autophagy act as cell survival mechanisms in most cases and are reported to play protective roles in pulmonary infection and inflammatory diseases [22]. Interestingly, mitophagy might also be cytotoxic if it is excessively induced to degrade even functional mitochondria [23,24]. In addition, emerging studies found that several pathogens, such as hepatitis B virus or hepatitis C virus, can adapt to autophagy induction, or even employ autophagy machinery to facilitate their replication [25,26,27]. The length of airway epithelial cilia can also be regulated by autophagic degradation (ciliophagy) [12]. Cilia shortening, mediated by ciliophagy, can impair the clearance ability of the airway against invading pathogens, thereby exacerbating pulmonary infection [28,29]. The double-edged sword role of autophagy in pulmonary diseases might be attributed to different cell types and different types of diseases. 

## 3. The Protective Roles of Autophagy in Acute Lung Injury (ALI)

**Figure 2:**
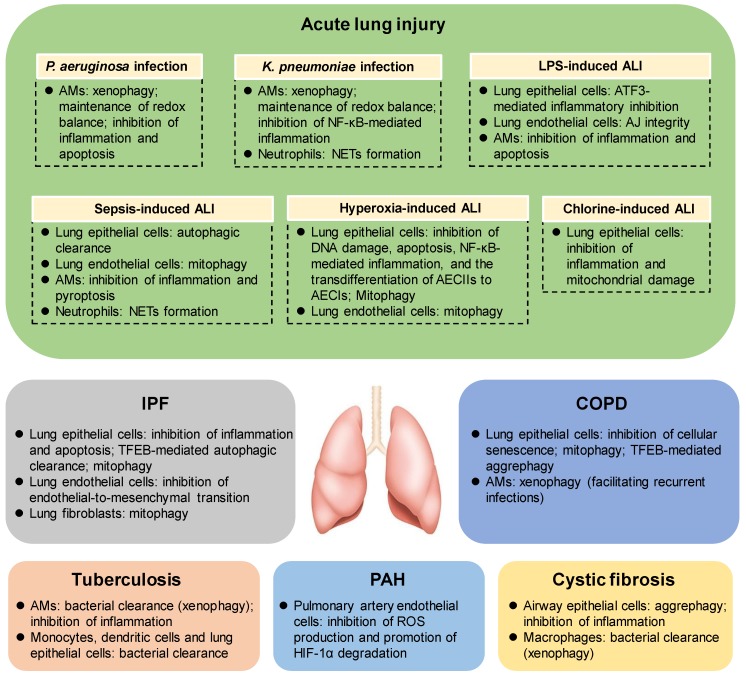
The protective mechanisms of autophagy in lung diseases. Autophagy may provide a protective role in the pathogenesis of various lung diseases (including ALI, IPF, COPD, tuberculosis, PAH, cystic fibrosis, etc.), through regulating diverse biological events, including inflammatory response, redox balance, DNA damage repair, apoptosis, pyroptosis, cellular senescence, NETs formation, mitochondrial homeostasis, pathogen or aggresome clearance, etc. ALI—acute lung injury; IPF—idiopathic pulmonary fibrosis; COPD—chronic obstructive pulmonary disease; PAH—pulmonary arterial hypertension; TFEB—transcription factor EB; AMs—alveolar macrophages; NETs—neutrophil extracellular traps; AJ integrity—adherens junctional integrity; *P. aeruginosa*—*Pseudomonas aeruginosa*; *K. pneumoniae*—*Klebsiella pneumoniae*; LPS—lipopolysaccharide.

Acute lung injury (ALI) is a common and severe clinical syndrome with high morbidity and mortality [7]. ALI is characterized with increased alveolar–capillary permeability, noncardiogenic pulmonary edema, neutrophil recruitment and diffuse alveolar damage, and represents a major cause of acute respiratory failure [11,30]. The intrapulmonary inflammatory response with the release of proinflammatory cytokines could be observed before and during the process of ALI, and has been intensively investigated recently [30,31]. However, the mechanisms underlying the pathogenesis and resolution of ALI remain largely unclear. Accumulating evidence suggests that autophagy is stimulated in response to diverse stimuli of ALI, including bacterial infection, lipopolysaccharide (LPS), sepsis, hyperoxia, and chlorine, etc. [32]. In addition, the loss of autophagy-related (ATG) genes, such as *Atg7*, *Atg5* and *Atg4b*, significantly aggravates the development of ALI in mice [11,32], suggesting that autophagy may exert protective effects for the initiation and progression of ALI in certain contexts (Figure 2).

### 3.1. The Protective Roles of Autophagy in Bacteria–Induced ALI

*Pseudomonas aeruginosa* (*P. aeruginosa*), an opportunistic Gram–negative human pathogen, was reported to induce autophagosome formation and subsequent autolysosomal degradation in alveolar macrophages (AMs), which are known as part of the first line of host defense in the lung [33]. The *P. aeruginosa*-induced autophagy is partially mediated by the Annexin A2 (ANXA2)–Akt1–mTOR–ULK1/2 and Beclin-1–ATG7–ATG5 signaling pathways [33,34]. *Anxa2* knockout mice exhibit elevated inflammatory cytokines, decreased bacterial clearance, increased lung injury and mortality [34]. How autophagy enhances host defense against *P. aeruginosa* remains largely to be investigated. We have recently found that following *P. aeruginosa* infection, toll-like receptor 2 (TLR2) initiates the phagocytic process in AMs and activates the *Src* kinase Lyn, which in turn delivers bacteria to lysosomes for degradation through xenophagy [35]. In addition to Lyn, the Wnt5A–Rac1–Disheveled pathway is also required for inducing xenophagy in AMs [36]. We also reported that regulation of redox balance and inflammatory response is involved in autophagy-mediated eradication of *P. aeruginosa*. *Atg7* deficiency promotes the release of reactive oxygen species (ROS) but limits NO production through inhibiting JAK2/STAT1α/NOS2 signaling, leading to the intracellular redox imbalance, elevated inflammatory cytokines, enhanced apoptosis of AMs, exaggerated lung infection and aggravated lung injury in mice [37]. Interestingly, *P. aeruginosa*-mediated autophagy induction and inflammasome activation can be mutually regulated to subtly orchestrate the host defense. For example, *P. aeruginosa* infection triggers protective autophagy by activating TLR4-TRIP signaling in bone marrow-derived macrophages (BMDMs). Meanwhile, the NLRC4 inflammasome can be activated, leading to caspase-1-mediated TRIF cleavage, and subsequent autophagy inhibition, thereby reducing bacterial clearance [38]. Autophagy, in turn, abrogates the activation of NLRC4 inflammasome by selectively removing damaged mitochondria (mitophagy) in BMDMs, leading to increased bacterial clearance [39]. Thus, autophagy induction and NLRC4 inflammasome activation may constitute a negative feedback loop in BMDMs following *P. aeruginosa* infection, which might facilitate the development of novel therapeutic options for the treatment of *P. aeruginosa* infection. However, whether this negative feedback loop is present in *P. aeruginosa*-infected AMs remains to be further investigated. 

*Klebsiella pneumoniae* (*K. pneumoniae*) is another Gram-negative bacterium that can activate the autophagy process in AMs [40]. It has been reported that *Atg7* deficiency significantly elevates the levels of inflammatory cytokines and superoxide, leading to increased susceptibility to *K. pneumoniae* infection in mice, suggesting that ATG7-mediated autophagy may represent a major resistance mechanism to *K. pneumoniae* infection [40]. Further study found that ATG7 can directly bind phosphorylated IκBα (p-IκBα). In *Atg7*–deficient AMs with *K. pneumoniae* infection, the binding of p-IκBα switches from ATG7 to ubiquitin, leading to the ubiquitin-mediated degradation of IκBα, activation of NF-κB, intensified inflammation, and decreased bacterial clearance [41]. Similar to *P. aeruginosa* infection, the TLR2–Lyn– or Wnt5A–Rac1–Disheveled-mediated xenophagy in AMs also contributes to the degradation and clearance of *K. pneumoniae* [35,36]. In addition to AMs, neutrophils also play important roles in the anti-bacterial host defense in the lung. In response to bacterial infection, the recruited neutrophils can release decondensed chromatin fibrils to form neutrophil extracellular traps (NETs) in a highly oxidative milieu, in order to trap, neutralize, and destroy microbes extracellularly [42]. It has been reported that TRPM2–AMPK–p38– or Mincle–mediated induction of autophagy is required for NETs formation following *K. pneumoniae* infection in a ROS–dependent or independent manner, respectively [43,44]. Future studies are needed for understanding the molecular mechanism underlying autophagy–regulated NETs formation during bacterial infection. 

### 3.2. The Protective Roles of Autophagy in LPS–Induced ALI

The outer membrane of Gram–negative bacteria is composed predominantly of LPS (also known as endotoxin), which is a pathogen-associated molecular pattern (PAMP) that enables the recognition of bacterial invasion and activates innate immune system [45]. It has been reported that LPS stimulation can regulate autophagy in lung epithelial cells, pulmonary endothelial cells and AMs. For example, LPS induces autophagy in mice lung tissues and bronchial epithelial cells. *Atg4b* deficiency significantly increases the susceptibility of the lung to LPS–mediated injury by impairing ATF3 activity, suggesting a protective role of autophagy in LPS–induced lung injury [46]. The LPS–induced protective autophagy may be due to the involvement of endoplasmic reticulum (ER) stress [47]. Interestingly, LPS was also reported to inhibit autophagy through TLR4– or AMPK inactivation–mediated mTOR activation in bronchial or alveolar epithelial cells [48,49]. *MTOR* knockdown, AMPK activation or autophagy stimulation significantly attenuates LPS-induced airway inflammation and injury, suggesting that autophagy functions as a protective mechanism to LPS–induced lung injury [48,49]. The inconsistent effects of LPS on the induction of autophagy may be due to different cell types and different sources of LPS. Despite this inconsistency, it can be concluded that autophagy in general confers a cytoprotective role in LPS–induced lung injury.

In addition to lung epithelial cells, LPS also induces autophagy in pulmonary endothelial cells. The inhibition of autophagy by si*ATG5*, si*ATG7* or chloroquine markedly reduces the permeability of human pulmonary microvascular endothelial cells and attenuates LPS-induced lung injury in mice, in part through restricting the injury of lung microvascular barrier, suggesting a protective role of autophagy in LPS–induced lung injury [50]. Mechanistically, RAB26, a newly identified small GTPase, can induce autophagic degradation of active SRC and the resultant CDH5 dephosphorylation, leading to the maintenance of lung vascular permeability and the protection of adherens junctional integrity in LPS–induced ALI [51]. In contrast, it was reported in another recent study that the inhibition of autophagy by 3-methyladenine (3-MA) significantly disrupts the endothelial barrier in human pulmonary artery endothelial cells and ameliorates lung vascular injury upon LPS treatment, suggesting that autophagy promotes LPS–induced lung injury [52]. This contradictory outcome of autophagy in LPS–induced lung injury might be due to the use of different autophagy inhibitors in different types of endothelial cells. 

LPS was also reported to induce autophagy in macrophages during LPS treatment. In response to LPS stimulation, the activated calcium/calmodulin-dependent protein kinase Iα (CaMKIα) can phosphorylate AMPK to form the CaMKIα–AMPK–ATG7 complex that contributes to autophagy induction in an mTOR–independent manner. The CaMKIα–AMPK–ATG7 signaling-mediated autophagy markedly attenuates LPS–induced lung neutrophilic inflammation [53]. Stimulation of autophagy by BML-111, a lipoxin A4 receptor agonist, significantly inhibits apoptosis, reduces the levels of proinflammatory cytokines, and ameliorates the LPS–induced lung injury [54]. These studies suggest that autophagy in macrophages confers the resolution of LPS–induced ALI, and may represent a potential therapeutic target.

### 3.3. The Protective Roles of Autophagy in Sepsis–Induced ALI

Sepsis is a syndrome of excessive inflammatory response to infection with high morbidity commonly leading to ALI [55]. However, the pathogenesis of sepsis still remains largely unclear. Emerging evidence suggests a critical role of autophagy in preventing sepsis, and the modulation of autophagy may provide novel insights for the treatment of sepsis. For example, in the cecal ligation puncture (CLP)–-induced sepsis mice model, the levels of LC3-II, ATG5, and ATG7 are downregulated in the lung of mice with sepsis, suggesting that sepsis may suppress autophagy. Stimulation of autophagy using rapamycin or activated protein C (APC) results in reduced inflammation and attenuated lung injury [56]. Interestingly, another group found that the LC3-II level is markedly increased in the lung of septic mice. The increased LC3-II level is due to autophagosome accumulation caused by the retarded autophagosome–lysosome fusion. Transgenic mice overexpressing the *LC3* gene exhibit accelerated fusion of autophagosome with lysosome, and survive longer after CLP [57]. This study suggests that the role of autophagy in CLP-induced sepsis might depend on the autophagic flux: the preservation of autophagic flux is cytoprotective to sepsis, while autophagosome accumulation due to impaired autophagic flux may contribute to lung injury in the late stage of sepsis. The discrepancy in LC3-II level observed in the same sepsis model in these two studies may be due to the ignorance of the LC3-II/LC3-I ratio, or the different detection time periods.

Mitochondrial dysfunction is recognized as an important mediator of sepsis pathogenesis. It was recently shown that the deficiency of kinase MKK3 ameliorates mitochondrial injury by lowering ROS levels and stimulating mitophagy, and increases mitochondrial biogenesis in pulmonary endothelial cells, leading to reduced septic lung injury [58]. Although the mechanism underlying the upstream signaling for mitophagy initiation remains to be defined, mitophagy may help provide novel therapeutic window for the treatment of sepsis. In addition to mitochondrial quality control, autophagy can also regulate inflammasome during sepsis. In a *P. aeruginosa*-induced sepsis mice model, *Atg7* deficiency significantly intensifies inflammasome activation and provokes pyroptosis in AMs, leading to impaired pathogen clearance and aggravated lung injury [59]. In addition, autophagy was found to be activated in neutrophils from both patients who survived sepsis or septic mice. Interestingly, autophagy augmentation in neutrophils leads to the formation of NETs in order to protect mice from lethal sepsis [60]. 

Considerable efforts have been made to develop autophagy–modulating strategies for the treatment of sepsis. For example, miR-155 was found to induce autophagy by inhibiting transforming growth factor-β (TGF-β)-activated kinase-1-binding protein 2 (TAB2), resulting in the alleviation of inflammation in septic lung injury [61]. In addition, it has been reported that the clinically approved anthracyclines at low doses can effectively confer disease tolerance to severe sepsis in mice via activation of DNA damage response and the stimulation of autophagy pathways in the lung [62]. Notably, carbon monoxide (CO) administered at low physiologic doses was reported to promote the Beclin 1–dependent autophagy process in lung epithelial cells through mitochondrial ROS generation, thereby increasing the survival of septic mice [63,64]. These studies suggest that CO exhibits a protective effect on sepsis, supporting the potential therapeutic application of CO for sepsis treatment.

### 3.4. The Protective Roles of Autophagy in Hyperoxia–Induced ALI

Hyperoxia (high levels of oxygen) exposure is commonly used for critically ill patients, including those with acute respiratory distress syndrome and chronic obstructive pulmonary disease (COPD) [65]. However, prolonged hyperoxia treatment induces the generation of excessive ROS, DNA damage and inflammatory response, leading to ALI and even respiratory failure [66]. The injury of pulmonary epithelium and subsequent apoptotic cell death is one of the major effects of hyperoxia [66]. It has been reported that hyperoxia upregulates ATG7, and induces LC3 turnover and autophagosome formation by activating c-Jun N-terminal kinase (JNK). Under hyperoxia, LC3 can interact with Fas by associating with caveolin-1 in lipid rafts to prevent apoptosis facilitating the survival of lung epithelial cells [67]. The hyperoxia–induced increased interaction of LC3 with Fas is due to the dissociation of LC3 with p62, an autophagic adaptor linking ubiquitinated substrates to the autophagy pathway for degradation [68]. Hyperoxia–mediated LC3 activation was also found to promote the accumulation of surfactant protein C (SP-C) in type II alveolar epithelial cells (AECIIs) and inhibit the transdifferentiation of AECIIs to type I alveolar epithelial cells (AECIs) [69,70]. In addition, hyperoxia–-induced ROS accumulation causes DNA damage in lung epithelial cells, which could be repaired by 8-oxoguanine-DNA glycosylase (OGG-1). Through regulating *ATG7* promoter, OGG-1 links DNA damage with autophagy in stimulating NF-κB–mediated inflammatory response to protect hyperoxia–induced epithelial injury [71]. Hyperoxia also causes a morphological change in mitochondria accomplished with increased expression of mitophagy–associated markers (PINK1 and PARK2) in lung epithelial cells, implying that mitophagy might play a role in protecting epithelial cells from hyperoxia–induced injury [72]. It is worth noting that the hyperoxia–induced ROS accumulation, mitochondrial damage and autophagy were also observed in pulmonary endothelial cells [73,74]. It has recently been reported that PINK1–mediated mitophagy is required for the ability of pulmonary endothelial cells to resist to hyperoxia [75]. It remains to be investigated whether autophagy functions in other types of lung cells under hyperoxia.

### 3.5. The Protective Roles of Autophagy in Chlorine–Induced ALI

Chlorine (Cl_2_), which is extensively used in industrial applications worldwide, is a common toxic inhalant [76]. Cl_2_ inhalation, depending on the dose and duration of exposure, may cause ALI and respiratory failure, and represents a significant public health problem [77]. Cl_2_ exposure to lung epithelial cells leads to mitochondrial dysfunction and ROS accumulation, which might be a major cause of lung injury [77]. Interestingly, autophagy can be induced to prevent mitochondrial damage, decrease inflammation, and ameliorate Cl_2_ toxicity [78]. This study suggests a protective role of autophagy in Cl_2_–induced lung injury, and implies that autophagy might represent a potential therapeutic target for the treatment of toxic Cl_2_ exposure. However, it lacks evidence of mitophagy in maintaining mitochondrial homeostasis, which merits further investigation. Moreover, autophagic alterations in Cl_2_–challenged pulmonary endothelial cells or AMs and their underlying mechanisms may also be critical to prevent lung injury, and remain poorly defined.

## 4. The Protective Roles of Autophagy in Idiopathic Pulmonary Fibrosis (IPF)

Idiopathic pulmonary fibrosis (IPF) is a chronic and fatal lung disease of unknown cause characterized by chronic lung inflammation, diffuse alveolar damage, the accumulation of fibroblasts and myofibroblasts, abundant collagen deposition and extracellular matrix proteins [79]. Decreased LC3-II expression and mTOR overactivation were observed in alveolar epithelial cells in bleomycin–induced pulmonary fibrosis mice model, as well as lung tissues from IPF patients compared to normal counterparts, suggesting impaired autophagic activity in IPF [80,81,82,83]. The compromised autophagy is due, in part, to the activation of IL-17A in lung epithelial cells during the fibrotic process. IL-17A stimulation activates the PI3K-glycogen synthase kinase 3β (GSK-3β) signaling pathway to inhibit Bcl-2 degradation, leading to the suppression of autophagy. Neutralization of IL-17A effectively induces autophagy, enhances collagen degradation, and decreases bleomycin–induced pulmonary fibrosis [84,85]. Moreover, *Atg4b*–deficient mice display reduced autophagy induction, increased inflammatory response, augmented apoptosis and hyperproliferation of alveolar and bronchiolar epithelial cells, thereby increasing collagen accumulation and exaggerating bleomycin–induced pulmonary fibrosis [86]. In addition, *Atg7* deficiency in endothelial cells impairs autophagic flux, upregulates TGF-β signaling, and promotes the endothelial-to-mesenchymal transition for fibroblast formation, leading to more extensive and severe fibrosis in bleomycin–challenged mice [87]. These studies suggest a protective role of autophagy in IPF. 

Indeed, stimulation of autophagy by rapamycin (an mTOR inhibitor promoting autophagic flux) significantly inhibits the fibrotic phenotype in bleomycin-induced pulmonary fibrosis. However, this protective effect of rapamycin can be partially reversed by chloroquine (an inhibitor of autolysosome formation blocking autophagic flux) [80,81,84,88]. Our recent findings suggest that bleomycin can directly bind ANXA2 in lung epithelial cells to prevent the translocation of transcription factor EB (TFEB) into the nucleus, leading to TFEB inactivation and impeded autophagic flux, thereby inducing pulmonary fibrosis. Pharmacological activation of TFEB using Torin 1 accelerates autophagic flux and significantly ameliorates bleomycin–induced pulmonary fibrosis [83]. These studies suggest that the autophagic flux might be inhibited to facilitate fibrotic progression in lung endothelial and epithelial cells. Further studies are required to investigate the upstream mechanisms by which autophagic flux is dysregulated in lung endothelial and epithelial cells in IPF. 

Autophagy alteration in fibroblasts has also been reported to be critical in human IPF pathogenesis. Interestingly, human IPF fibroblasts show reduced autophagy induction and decreased autophagic flux, due to mTOR activation or reduced FoxO3a–mediated LC3 transcription [89,90,91]. The defective autophagy is required for maintaining the cell death-resistant phenotype for the fibroblasts on collagen-rich matrix [90,91]. Considering the profibrotic role of autophagy in IPF fibroblasts, the use of autophagy activators for IPF treatment should be re-evaluated in a context–specific manner.

Emerging evidence reveals the critical roles of deregulated mitochondrial homeostasis in AECIIs, fibroblasts or AMs in the pathogenesis of IPF. For example, it was reported that dysmorphic and dysfunctional mitochondria are accumulated in AECIIs in the lungs of IPF patients [92]. The impaired mitochondria in AECIIs are associated with decreased levels of PINK1 and defective mitophagy in AECIIs. *PINK1*–deficient mice exhibit deregulated mitochondrial homeostasis and development of pulmonary fibrosis [92]. The expression of PARK2, another mitophagy–associated protein, is downregulated in the lung fibroblasts of IPF patients. *PARK2* deficiency aggravates bleomycin–induced pulmonary fibrosis in mice through promoting PDGFR-PI3K-Akt-mediated myofibroblast differentiation and proliferation [93]. Pirfenidone, an FDA–approved agent for IPF treatment, exerts its anti-fibrotic effect partially by inducing PARK2–mediated mitophagy and inhibiting myofibroblast differentiation [94]. Different from the protective role of mitophagy in AECIIs or fibroblasts for IPF, mitophagy is increased in IPF AMs and is required for the development of pulmonary fibrosis. During fibrosis, Akt1 in AMs is activated to induce the generation of mitochondrial ROS, leading to stimulation of PARK2–mediated mitophagy [95]. The Akt1–mediated mitophagy induction contributes to apoptosis resistance of AMs, enables the expression of macrophage–derived TGF-β1, and ultimately promotes fibroblast differentiation and progression of pulmonary fibrosis [95]. Given the contrary effects of mitophagy of different cell types in IPF pathogenesis, the manipulation of cell type–specific mitophagy, rather than global mitophagy, may achieve better therapeutic outcome for IPF treatment.

## 5. The Protective and Deleterious Roles of Autophagy in COPD

COPD is a pulmonary disorder characterized by excessive inflammation and airway obstruction (i.e., chronic bronchitis and emphysema) [96]. Cigarette smoke (CS) remains the key risk factor for COPD worldwide [96]. The molecular mechanisms underlying COPD pathogenesis remain incompletely understood. It has been shown that the expression of ATG proteins, such as LC3, is increased in lung tissues from COPD patients and mouse lung tissues subjected to CS exposure, suggesting an increase of autophagosome formation in COPD [97,98]. The increased autophagosome formation is correlated with a cumulative increase in autophagic flux, suggesting that the autophagy pathway in lung epithelial cells is activated in COPD [28,99]. The increased activity of autophagy caused by CS exposure is at least partially due to the decreased histone deacetylase (HDAC) –mediated Egr-1 inhibition, elevated PGF–JNK1–p38–TSC2–mediated mTOR inhibition, or upregulation of oxidative stress-induced growth inhibitor 1 (OSGIN1) [98,100,101,102]. In response to CS exposure, LC3 dissociates from the extrinsic apoptotic factor Fas, leading to apoptotic cell death of lung epithelial cells for emphysema progression [103]. The activation of autophagy is also observed in particulate matter (PM) –induced experimental COPD model, and *Atg7* deficiency protects mice from PM–induced COPD [104]. In addition to lung epithelial cells, increased autophagy was also observed in CS-treated neutrophils. CS exposure induces autophagic cell death of neutrophils by activating PAFR–HMGB1–Beclin-1–Bcl-2 signaling, leading to the development of emphysema [105]. These studies indicate that autophagy stimulation with increased autophagic flux, either in lung epithelial cells or neutrophils, contributes to the development of COPD. 

Interestingly, autophagy has been reported to regulate bronchial epithelial cell senescence in CS–induced senescence–associated COPD. CS exposure leads to autophagy inhibition in COPD patients, which might be, in part, due to the activation of the SIRT6–IGF–Akt–mTOR signaling pathway [106,107]. Autophagy inhibition by 3-MA results in increased senescence in human bronchial epithelial cells, whereas autophagy activation by Torin 1 significantly inhibits cell senescence, indicating that the insufficient autophagy accelerates bronchial epithelial cell senescence in COPD [106]. The CS–impaired autophagy is characterized by the enhanced formation of aggresome and resultant insufficient autophagic clearance (impaired aggrephagy) [106,108,109]. A possible mechanism of CS–impaired autophagy is the perinuclear aggresome sequestration of TFEB, the master regulator of autophagy. Activation of TFEB using gemfibrozil significantly decreases CS–induced formation of aggresome, resulting in the mitigation of COPD progression [110]. These studies suggest that CS exposure promotes the accumulation of aggresome bodies and consequent autophagy impairment, which accelerates bronchial epithelial cell senescence and exacerbates the development of COPD.

The pathogenesis of CS–induced COPD is also associated with elevated levels of ROS caused by mitochondrial damage [111]. PARK2 deficiency results in increased mitochondrial damage, enhanced ROS accumulation, and accelerated senescence of lung epithelial cells under CS exposure, suggesting that CS–induced PARK2–mediated mitophagy may attenuate cellular senescence and inhibit the progression of COPD [111,112]. However, a study from another group indicates that PINK1–regulated mitophagy promotes necroptosis and cell death in lung epithelial cells, thereby contributing to COPD development [23]. The different outcomes of mitophagy in regulating senescence or necroptosis probably depend on the injury degree in response to CS [23,111]. In addition to damaged mitochondria, CS–induced autophagy also regulates cilia length by selective consumption of cilia components (ciliophagy) in respiratory epithelial cells in COPD pathogenesis [28,29]. In contrast to mitophagy and ciliophagy, CS exposure leads to autophagy inhibition and xenophagy impairment in AMs. In smokers’ AMs, the autophagy degradation is defective as evidenced by the accumulation of both LC3 and p62, which may explain the clinical issue of recurrent infections for smokers [113]. 

Collectively, it seems that different autophagy machineries are involved in COPD pathogenesis, and the roles of autophagy in COPD pathogenesis vary in different reports. One possibility is that autophagy machineries in different types of lung cells are differentially regulated. In addition, the period of CS exposure in animals or the stages in humans might also be critical to the roles of autophagy in CS–induced COPD. Further studies are needed to decipher the precise roles of autophagy in COPD.

## 6. The Protective Roles of Autophagy in Tuberculosis

Extensive studies have demonstrated the critical roles of autophagy in the pathogenesis of tuberculosis caused by *Mycobacterium tuberculosis* (Mtb) infection. Mtb could interfere with the fusion of autophagosome with lysosome to prevent autophagosome maturation and subsequent autolysosomal degradation in macrophages [114,115]. Stimulation of autophagy by rapamycin, IFN-γ or vitamin D promotes autophagic flux, enabling autophagy–mediated clearance of Mtb [114,116]. In addition to suppressing Mtb growth, autophagy also contributes to the resolution of damaging inflammation [117]. Interestingly, autophagy in monocytes is also involved in Mtb defense, and induction of autophagy in monocytes could enhance the antimicrobial activity against Mtb [118,119]. Moreover, the Mtb–eradicating role of autophagy was observed in dendritic cells and lung epithelial cells [120,121,122]. However, a recent study shows that myeloid cell–specific deficiency of *Atg5*, but not other ATG genes (including *Atg3*, *Atg7*, *Atg12*, *Atg14L* and *Atg16L1*), significantly provokes Mtb infection and hampers the survival of infected mice [123]. This study suggests that the canonical autophagy pathway may not play a major role in the pathogenesis of tuberculosis. Instead of autophagy induction, Atg5 functions in a protective manner for Mtb infection by preventing polymorphonuclear cell (PMN)–mediated immunopathology [123]. Therefore, a more in-depth evaluation of the role of autophagy in tuberculosis pathogenesis is needed.

## 7. The Protective Roles of Autophagy in Cystic Fibrosis (CF)

Cystic fibrosis (CF) is a life–threatening lung disease caused by a loss-of-function mutation of cystic fibrosis transmembrane conductance regulator (CFTR, F508del) [124,125]. It has been reported that CFTR deficiency causes defective autophagic flux in both human airway epithelial cells and nasal biopsies from CF patients, leading to the formation of aggresome through the production of ROS, upregulation of tissue transglutaminase (TG2), sequestration of the class III PI3K complex and subsequent accumulation of p62 [126,127]. This disruption of autophagic clearance also heightens the inflammatory response in CFTR–mutant cells [128]. In addition to the airway epithelial cells, defective autophagic degradation was also observed in macrophages with CFTR mutation. The decreased autophagic clearance subverts the bactericidal function of macrophages, consequently resulting in pathogen infection, such as *Nontuberculous mycobacteria* (NTM) and *Burkholderia cenocepacia* (*B. cepacia*) [129,130]. Induction of autophagy by rapamycin or clearance of aggresome by p62 deletion could markedly enhance the elimination of pathogens and ameliorate the associated inflammation [130,131]. Together, these studies suggest that autophagy is a survival mechanism in the pathogenesis of CF, and pharmacological induction of autophagy might be a promising strategy to delay CF progression.

## 8. The Protective Roles of Autophagy in Pulmonary Arterial Hypertension (PAH)

Pulmonary arterial hypertension (PAH) is a lethal syndrome characterized by elevated pulmonary arterial pressure with unclear etiology [132]. Hypoxia is known as a common cause of PAH. It has been reported that human lungs with PAH reveal elevated LC3B levels and increased autophagosomes compared to normal lungs. In addition, autophagy induction is promoted following hypoxia treatment in human pulmonary artery endothelial cells (PAECs). In a chronic hypoxia–induced PH model, LC3B knockout mice show apparent PAH phenotypes relative to wild-type mice [133]. These results suggest a protective function of autophagy in PAH pathogenesis. The stimulation of autophagy was also observed in pulmonary artery smooth muscle cells (PASMCs) in a rat PAH model induced by monocrotaline or hypoxia. Paradoxically, the inhibition of autophagy by chloroquine or κ-opioid receptor exerts beneficial effects for PAH [134,135], implying that autophagy may contribute to the pathogenesis of PAH. The various roles of autophagy in PAH pathogenesis might be explained by the different cell types, approaches and models used in these studies.

## 9. Conclusions and Perspectives

Accumulating evidence demonstrates that autophagy is involved in the regulation of diverse biological functions, such as inflammatory response, redox balance, DNA damage repair, apoptosis, and necroptosis in different cell types in the lung, and thus plays crucial roles in pulmonary infection and inflammatory diseases, including ALI, IPF, COPD, tuberculosis, PAH, CF, etc. (Figure 2). Autophagy is initially known as a protective process in the pathogenesis of most lung diseases. Recent findings also support the notion that autophagy may promote the pathogenesis of lung diseases in certain contexts. The diverse roles of autophagy in lung disease pathogenesis might be due to the different types of lung diseases (ALI, IPF, COPD, tuberculosis, PAH, CF, etc.), the diverse stressors for the etiology (infection, CS exposure, the stimulus intensity, etc.), the various cell types in the lung (epithelial cells, endothelial cells, fibroblasts, neutrophils, AMs, etc.), and the different mechanisms underlying disease initiation and progression (cell death such as apoptosis and necroptosis, cellular senescence, fibroblast differentiation, DNA damage, etc.). In addition, the analysis methods, experimental approaches, reagents, and models with different cells and animals (e.g., age, sex) all contribute to the variations in the laboratories. Furthermore, selective autophagy, such as mitophagy, xenophagy, aggrephagy, and ciliophagy, has recently attracted much attention in the pathogenesis of human lung diseases. It remains unclear how cells orchestrate nonselective autophagy and selective autophagy during disease initiation and progression, and whether nonselective autophagy cross-talks with selective autophagy [22]. Moreover, the lack of autophagy inhibitors to specifically target nonselective autophagy and selective autophagy in a given lung cell type also remains a major challenge for therapeutic intervention. Finally, some of the previous studies (especially those in vivo studies) only examined LC3-II accumulation which might be a result of the activation or inhibition of autophagic flux–mediated degradation. Therefore, the non-unified interpretation of autophagy activation and suppression remains a major problem for the evaluation of the exact roles of autophagy in pathological or therapeutic aspects. Careful consideration of the autophagy activity is needed to achieve a better and deeper understanding of the role of autophagy in lung disease pathogenesis for the development of potential therapeutic strategies.
